# *Helicobacter pylori* regulates stomach diseases by activating cell pathways and DNA methylation of host cells

**DOI:** 10.3389/fcell.2023.1187638

**Published:** 2023-05-04

**Authors:** Yue Xi, Xiao-Li Zhang, Qing-Xin Luo, Hai-Ning Gan, Yu-Shi Liu, Shi-He Shao, Xu-Hua Mao

**Affiliations:** ^1^ School of Medicine, Jiangsu University, Zhenjiang, China; ^2^ Department of Clinical Laboratory, The Affiliated Yixing Hospital of Jiangsu University, Wuxi, China

**Keywords:** *Helicobacter pylori*, signaling pathway, apoptosis, DNA methylation, gastric cancer

## Abstract

One of the most prevalent malignant tumors of the digestive tract is gastric cancer (GC). Age, high salt intake, *Helicobacter pylori* (*H. pylori*) infection, and a diet deficient in fruits and vegetables are risk factors for the illness. A significant risk factor for gastric cancer is infection with *H. pylori*. Infecting gastric epithelial cells with virulence agents secreted by *H. pylori* can cause methylation of tumor genes or carcinogenic signaling pathways to be activated. Regulate downstream genes’ aberrant expression, albeit the precise mechanism by which this happens is unclear. Oncogene, oncosuppressor, and other gene modifications, as well as a number of different gene change types, are all directly associated to the carcinogenesis of gastric cancer. In this review, we describe comprehensive *H. pylori* and its virulence factors, as well as the activation of the NF-κB, MAPK, JAK/STAT signaling pathways, and DNA methylation following infection with host cells via virulence factors, resulting in abnormal gene expression. As a result, host-related proteins are regulated, and gastric cancer progression is influenced. This review provides insight into the *H. pylori* infection, summarizes a series of relevant papers, discusses the complex signaling pathways underlying molecular mechanisms, and proposes new approach to immunotherapy of this important disease.

## Introduction

The third most common cause of cancer-related death worldwide, gastric cancer (GC) is one of the most prevalent malignant tumors. The prevalence of GC varies greatly among various geographical areas (Machlowska et al., 2020; Petryszyn et al., 2020). Its growth and evolution are multi-year, multi-stage processes that continue to be a problem for global health today (Gao et al., 2018).

The most significant risk factor for GC is *Helicobacter pylori* (*H. pylori*), which is one of the most prevalent infectious organisms in humans globally (Noto and Peek, 2012). *H. pylori* is categorized by the World Health Organization (WHO) as a class 1 carcinogen (Crowe, 2019). Numerous virulence factors produced by *H. pylori* have the potential to disrupt intracellular signaling pathways in the host and lower the threshold for tumor transformation. In addition, gastric cancer, the *H. pylori* infection can also cause other stomach diseases, including gastric ulcer, duodenal ulcer, stomach atrophy and other diseases. The *H. pylori* infection is strongly associated with major gastritis and multifocal atrophy, and testing for *H. pylori* is another important component of screening for these diseases.

The main pathogenic agents in the *H. pylori* infection are CagA (cytotoxin-related gene A), its pathogenicity island (Cag PAI), and VacA (vacuolar cytotoxin A) (Wang F et al., 2014). The CagA protein and the Cag IV secretion system (T4SS) are encoded by 27–31 genes in the 40 kb Cag PAI DNA insertion element (Muller, 2012; Backert et al., 2015). The three proteins CagL, CagI, and CagH are parts of the T4SS subcomponents and are all necessary for Cag T4SS, which may be crucial in the development of the infectious contact between *H. pylori* and stomach epithelial cells. Through bacteria and epithelial cells, CagA T4SS transports CagA from connected *H. pylori* into host cells, through the passage of bacterial and epithelial membranes, CagA T4SS carries CagA from the associated into the host cells (Odenbreit et al., 2000; Fischer et al., 2001; Shaffer et al., 2011). It binds to the inside of cell membranes, causing the downstream signaling pathways to be activated by tyrosine phosphorylation at the N-terminal glutamate-proline-isoleucine-tyrosine-alanine (EPIYA) site (Wroblewski and Peek, 2016). Vacuolar cytotoxin A (VacA), an 88 kDa protein made up of the p33 and p55 protein subunits, can be secreted using the IV-type autotransporter and secretion system. This protein alters a number of things, including the permeability of the mitochondrial membrane, the vacuolation of host gastric epithelial cells, autophagy, apoptosis, and disruption of epithelial tight junctions. Additionally, it prevents lamina propria T lymphocyte activation and proliferation (Cover and Blanke, 2005; Palframan et al., 2012; Raju et al., 2012).

The release of virulence factors following the *H. pylori* infection of gastric epithelial cells can activate downstream signaling pathways and associated processes, including the nuclear factor κB (NF-κB) pathway and the cytokine-stimulated transduction (JAK-STAT) signaling system. Furthermore, these virulence factors have the ability to induce apoptosis and methylation of the relevant proteins, which can control the expression of a number of host proteins and influence the appearance and growth of GC.

## *Helicobacter pylori* infected host cells activate NF-κB signaling pathway

Gastric epithelial cells were colonized by *H. pylori*, which then activated the natural and NF-κB pathway (Maubach et al., 2021; Maubach et al., 2022). Inflammation is brought on by *H. pylori* CagA stimulating the NF-κB pathway and binding TAK1 to TRAFs, which in turn activates the IκB kinase (IKK) comple*x* (Brandt et al., 2005; Zhang et al., 2019). Protein modification and intracellular location control the formation of homologous dimers and heterodimers that both activate and inhibit transcription (Neumann and Naumann, 2007), the NF-κB family of transcription factors regulates immunological response, inflammatory response (Hayden and Ghosh, 2011), cell proliferation, differentiation, and genomic stability (Liu et al., 2017; Peng et al., 2020).

Following the *H. pylori* infection, GC cells secreted more IL-8 and IL-32. For the IL-8 gene to be transcribed, the essential transcription factor NF-κB must be activated by binding to either AP-1 or NF-IL6 (Yasumoto et al., 1992), in GC cells, AP-1 may take the place of NF-IL6 and work in conjunction with NF-κB to cause IL-8 gene transcription binding to form a complex (Aihara et al., 1997). Additionally, NF-κB increases the expression of the inflammatory cytokine IL-32, which in turn increases the expression of NF-κB. This occurs mostly through the CAG pathogenicity island (cagPAI)-positive (Sakitani et al., 2012). A crucial metalloproteinase is MMP-7, in a CAG pathogenicity island (cagPAI) -dependent way, causes a persistent inflammatory response and increases gastrin expression in gastric epithelial cells. Gastrin increases MMP-7 via triggering the NF-κB signaling pathway via the protein kinase C-dependent pathway linked to IκB kinase. Additionally, it stimulates the expression of the HB-EGF gene for epidermal growth factor and ectodomain shedding. (Bebb et al., 2003; Ogasa et al., 2003; Dickson et al., 2006; Yin et al., 2010). Additionally, a the *H. pylori* infection of the stomach causes the release of MMP-9, a matrix metalloproteinase (Hojo et al., 2000; Göõz et al., 2001), the intracellular kinases NIK and IKKs stimulate the NF-κB signaling pathway in gastric epithelial cells infected with cagPAI-positive *H. pylori*, controlling the production of MMP-9 (Maeda et al., 2000; Mori et al., 2003). The virulence factor urease and the effects of *H. pylori* on the expression and transcription levels of MUC in MUC genes (MUC5AC, MUC2, and MUC6) in gastric mucosa of Kato-III may upregulate the expression of chemokines and proinflammatory factors while downregulating the transcription of the MUC5AC gene in GC cells. On the other hand, the MUC5AC promoter has a κB cis-element, which reduces the activity of the promoter and lowers expression (Perrais et al., 2014). MUC1 is essential for controlling how negatively NLRP3 inflammasome activation affects immune cells. MUC1 expression rises following the *H. pylori* infection, which prevents NLRP3 from being activated by blocking the TLR/NF-κB-dependent signaling pathway. This, in turn, prevents the inflammatory response brought on by chronic the *H. pylori* infection (Ng et al., 2016; Zhang et al., 2022a).

**FIGURE 1:**
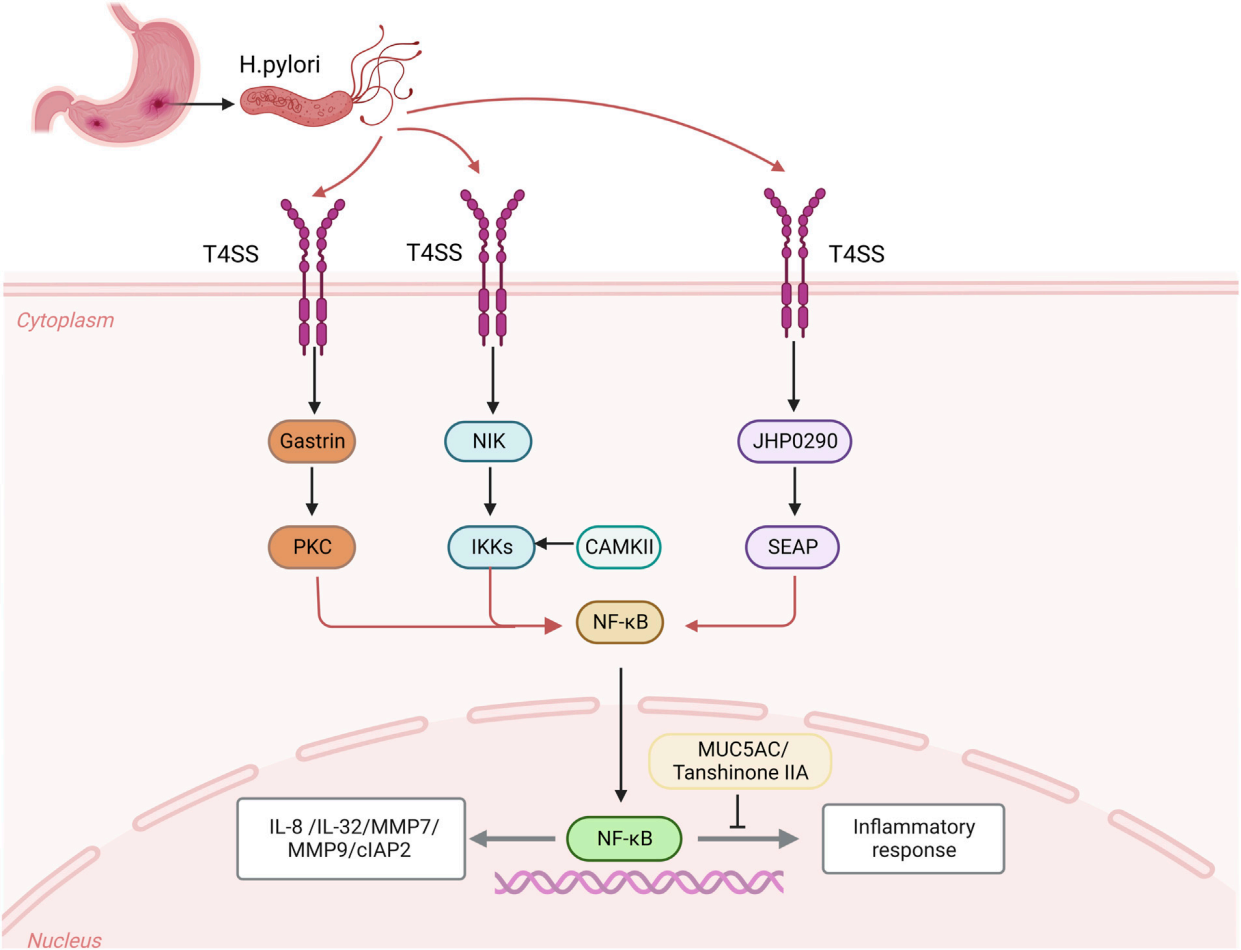
After the *Helicobacter pylori* infection, CagA is transferred from the attached *H. pylori* through the bacteria and epithelial cells to the host cell through the CagA-T4SS secretion system, which activates the expression of intracellular kinases NIK and gastrin as well as the homologues of JHP0290, and activates the activities of downstream intracellular kinases IKKs, PKC and SEAP, activate the NF-κB signaling pathway to regulate the expression of inflammatory cytokines IL-8 and IL-32, MMP7 and MMP9, and apoptosis inhibitor protein 2 (cIAP2). CAMKII and calmodulin can activate IKK complex and induce NF-κB signal. After the *H. pylori* infection, MUC5AC could bind to κB cil-element promoter, which caused decreases of MUC5AC promoter’s activity and expression of MUC5AC. The release of Tanshinone IIA after the *H. pylori* infection can effectively inhibit the expression of NF-κB nuclear protein and inhibit the inflammatory signal.

One of the primary proteins regulating apoptosis is inhibitor of apoptosis protein (IAP), and cIAP2 (inhibitor of Apoptosis protein 2) is crucial for the development of cancer (Chen et al., 2020). The advancement of AG/IM and the *H. pylori* infection are not the only conditions that are linked to the overexpression of cIAP2 in GC, the primary mechanism is that *H. pylori* upregulates the expression of cIAP2 by activating the NF-κB signaling pathway in a cagPAI dependent manner (Yoon et al., 2017). A homolog of HP0305 called JHP0290 can attach to different cell types and alter macrophage reactions (Pathak et al., 2013). Different *H. pylori* strains can express and release the JHP0290 homolog. NF-κB activation was seen in gastric epithelial cells that had been stimulated by JHP0290, which considerably increased the amount of alkaline phosphatase (SEAP) activity in GC cells in a dose-dependent manner and activated the NF-κB signaling pathway, which controls a number of cellular processes in cancer (Tavares and Pathak, 2015). Additionally, *H. pylori* stimulates the LIGHT pathway, a distinct group of receptors in the tumor necrosis factor superfamily, in addition to the canonical NF-κB signaling pathway, which necessitates functional T4SS (TNFSF). The main mechanism is that the CAG pathogenicity island (cagPAI), after infecting gastric epithelial cells with *H. pylori*, stimulates ligand binding to the LTβR produced by epithelial cells and draws in immune cells to increase chemokines and suppress the usual NF-κB signaling pathway. There is a close connection between the common and alternate pathways during the *H. pylori* infection (Mejias-Luque et al., 2017). The expression of CAMKII (Ca21/calmodulin dependent kinase II) is regulated by calmodulin, the IKK complex is activated by CAMKII and calmodulin, which triggers the NF-κB signaling pathway (Maubach et al., 2013). Additionally, after the *H. pylori* infection, several medical substances can help control how proteins are expressed. Tanshinone IIA can effectively inhibit the activation of the NF-κB signaling pathway, destroying the production of downstream inflammatory substances and effectively reducing the inflammatory response induced by *H. pylori*. After the *H. pylori* infection, the expression of nuclear NF-κB (p65) protein increases significantly (Chen et al., 2016) (Figure 1).

## *Helicobacter pylori* infected host cells activate ERK/MAPK signaling pathway

The MAPK signaling pathway can be activated by *H. pylori*-induced gastric epithelial cell growth and gene expression (Sebkova et al., 2004; Zhu et al., 2005; Ding et al., 2008). The mitogen-activated protein kinase (MAPK) family, which participates in signaling cascades and transmits extracellular signals to intracellular destinations, includes extracellular signal-regulated kinase 1/2 (ERK). In eukaryotic cells: ERK, JNK/stress-activated protein kinase, P38 MAPK, and ERK5 signal transduction pathways have all been found (Guo et al., 2020b). The extracellular signal-regulated protein kinase (ERK) cascade, which is typically controlled by the activation of cell-surface receptor tyrosine kinases (RTKS), is the most prevalent of these (Katz et al., 2007). A fundamental signal transduction system known as the MAPK signaling pathway controls cell growth, differentiation, and stress response (Plotnikov et al., 2011; Sabio and Davis, 2014; Guo et al., 2020b).

AUF1 is upregulated when *H. pylori* is present as a result of CagA-induced ERK pathway activation. GKN1 mRNA, a gastric tumor suppressor, can have its stability controlled by the ARE-binding protein AUF1. In light of this, it is possible that the CagA/p-ERK/AUF1 axis is crucial in the downregulation of the downstream AUF1 effector GKN1mRNA. Making it a GC oncogene (Guo et al., 2020a). Through the NF-κB signaling pathway, *H. pylori* can cause the expression of metalloproteinases, and through the ERK signaling pathway, it can control the expression of metalloproteinases. The *H. pylori* CAG pathogenicity island (cagPAI), which is implicated in GC metastasis, induces the ERK1/2 signaling pathway, which is responsible for the upregulation of MMP-1 production in gastric epithelial cells (Krueger et al., 2006; Jiang et al., 2014). The ERK1/2 signaling pathway activated by *H. pylori* also controls MMP-10. The CAGA-positive *H. pylori* strain damages the stomach epithelium by prompting gastric epithelial cells to produce and secrete active MMP-10 in GC cells, which in turn activates the tyrosine kinase receptor EGFR (Costa et al., 2016). Additionally, the MMP-10 expression caused by *H. pylori* was reduced by the red-orange pigment β-carotene, which *in vivo* can be converted to retinaldehyde, mostly by activating PPAR-γ and triggering its downstream target catalase. As a result, *H. pylori*-infected gastric epithelial cells have lower levels of ROS and the ERK signaling pathway, as well as lower levels of MMP-10 expression and *H. pylori*-associated GC incidence (Costa et al., 2016; Bae et al., 2021).

**FIGURE 2:**
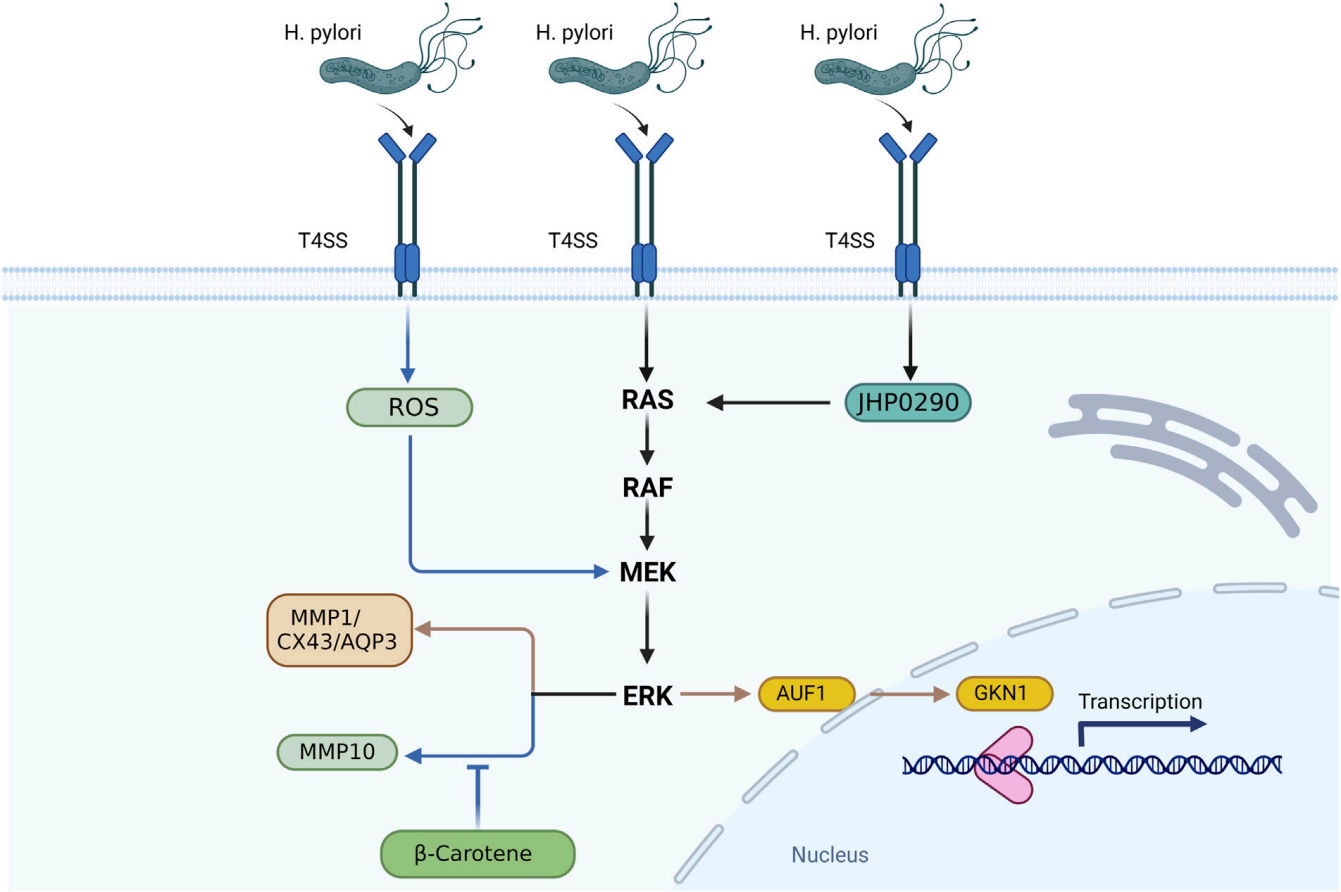
After the *H. pylori* infection, virulence factors are secreted into cells through the CagA-T4SS secretion system, activation of ROS and Ras/Raf/MEK proteins, activation of downstream ERK/MAPK signaling pathway, upregulate of AUF1 and control of GKN1 expression, ERK also control the expression of metal proteinases MMP1 and MMP10. β-carotene inhibits *H. pylori* induced the expression of MMP10, and the *H. pylori* virulence factor can upregulate Cx43 expression, and then activate the ROS-dependent ERK signaling pathway to reverse control Cx43 production and apoptosis. AQP3 is involved in the changes of cell behavior induced by *H. pylori* through the regulation of ERK pathway. JHP0290 released by *H. pylori* protein can activate the ERK pathway and regulate the proliferation of gastric epithelial cells.

The alteration in cell behavior brought on by *H. pylori* is mediated by AQP3. The expression of AQP3 in gastric cells is primarily controlled by the ERK signaling pathway, and reduction of AQP3 can inhibit the proliferation and migration of cancer cells generated by *H. pylori*. An essential membrane protein called Cxs controls the creation of intercellular channels, the interchange of signaling chemicals, and intercellular communication. Additionally, it plays a crucial role in intercellular communication (Vinken et al., 2006; Gemel et al., 2014). The virulence factor of *H. pylori*, the fact that VacA has no effect on the membrane protein Cx43’s mRNA level raises the possibility that VacA might promote Cx43 accumulation by preventing Cx43 degradation. The amount of GSH that is turned over in GC cells may be controlled by VacA released by *H. pylori* (Kimura et al., 2001); the GSH levels influence the ROS-dependent ERK signaling pathway’s activity, which controls the generation of Cx43 and apoptosis (Yahiro et al., 2015). Furthermore, *H. pylori* JHP0290 protein can not only activate the NF-κB pathway to participate in the inflammatory response, but also activate ERK MAPK in a dose-dependent manner to regulate the proliferation of gastric epithelial cells (Tavares and Pathak, 2015) (Figure 2).

## *Helicobacter pylori* infected host cells activate JAK/STAT signaling pathway

Through the JAK/STAT signaling system, the *H. pylori* infection of gastric epithelial cells can control cell growth and the production of associated proteins. An essential signal transduction system is the JAK/STAT (Janus kinase/Signal Converter and Activator of transcription) cascade (Khanna et al., 2015). To control the expression of the associated genes, JAK phosphorylates STAT, which dimerizes and travels to the nucleus through the nuclear envelope (Xin et al., 2020). A family of non-transmembrane tyrosine kinases is known as the JAK family. The JAK clan is Most members of the JAK family are JAK1, JAK2, JAK3, and Tyk2 (Cai et al., 2015). One of the most important activating transcription factors in the immune response is the STAT family, which is a downstream target of JAKs in the cytoplasm. There are seven people who make up the group: STAT1, STAT2, STAT3, STAT4, STAT5A, STAT5B, and STAT6 (Boengler et al., 2008; Yu et al., 2009), cell proliferation, stem cell self-renewal, and immunological responses are only a few examples of the physiological and cellular processes that JAK/STAT signaling is involved in that are connected to the beginning and development of disease (Aaronson and Horvath, 2002; Rawlings et al., 2004).

Human inter-trypsin inhibitor heavy chain 4 (ITIH4) is an acute phase response protein that is positively regulated by interleukin-6 (IL-6) (Piñeiro et al., 1999; Liu et al., 2016), and TIP-α (tumor necrosis factor-α-inducible protein), a newly identified membrane protein secreted by *H. pylori*, is a potent inducer of epithelial-mesenchymal transition (EMT). After the *H. pylori* infection, the secretion of ITIH4 and TIP-αmay encourage the production of IL-6 in GC cells, IL-6 can then trigger the expression of p-STAT3, which activates the STAT3 signaling pathway. The IL-6/STAT3 pathway may be activated by Tip-α and ITIH4 to speed up GC (Jove, 2000; Liu et al., 2016; Chen et al., 2017; Suganuma et al., 2021). TMEFF2 is a signaling transmembrane protein that interacts with two folliclestatin proteins and epidermal growth factor (Costa et al., 2010; Lin et al., 2011). TMEFF2 in the GC may not be regulated normally as a result of the *H. pylori* infection. The primary mechanism is that, in the early stages of the *H. pylori* infection, the overexpression of TMEFF2 in healthy gastric mucosa causes the production of SHP-1, a protein tyrosine phosphatase, which inhibits STAT3 activation. A long-lasting the *H. pylori* infection can activate the STAT3 signaling pathway, control STAT3 phosphorylation, and bind directly to the TMEFF2 promoter to suppress TMEFF2 production in the opposite direction (Sun et al., 2015).

**FIGURE 3:**
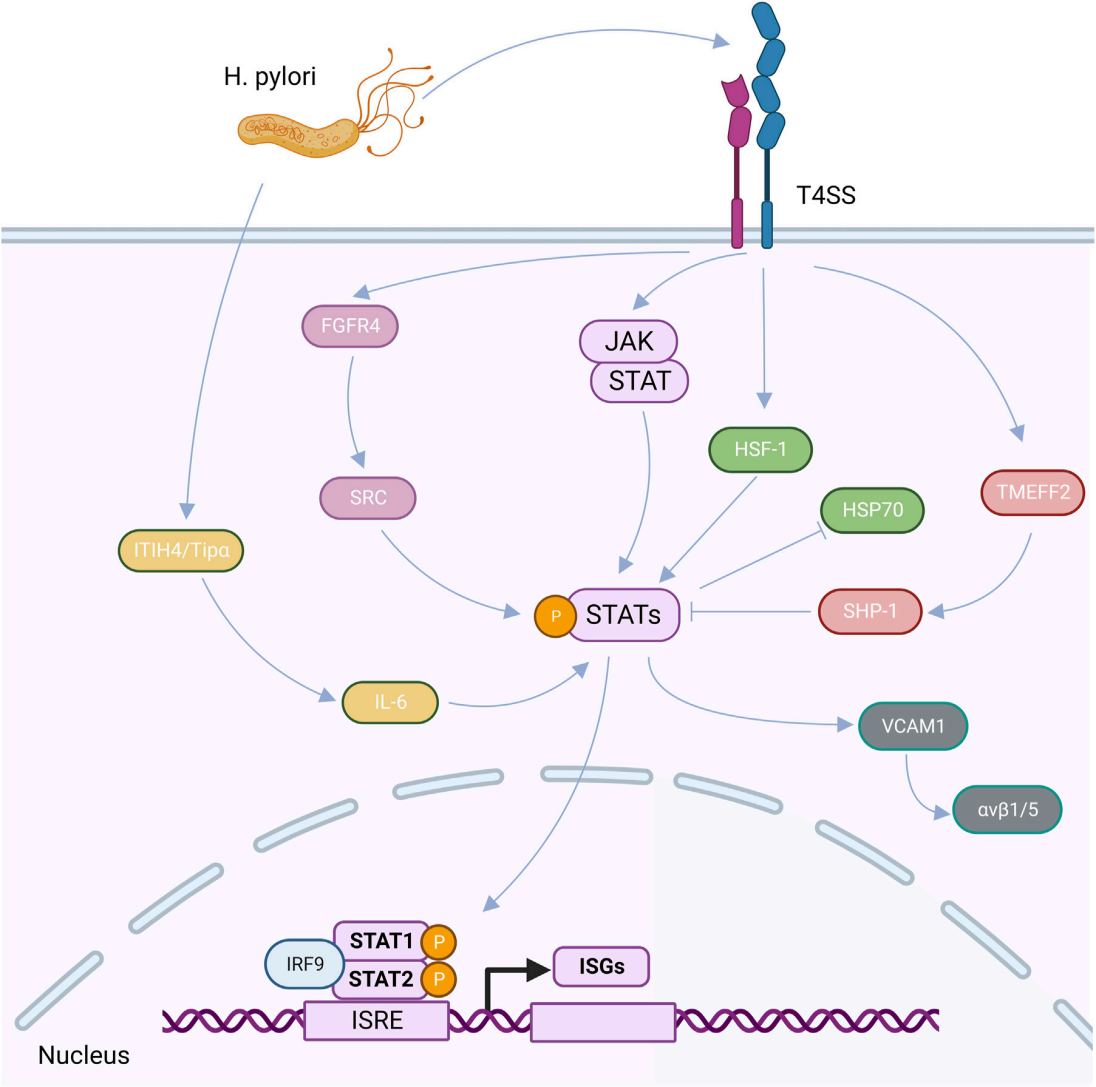
After the *H. pylori* infection, virulence factors can be secreted into cells through the CagA-T4SS secretion system, upregulated ITIH4 and TiP-α, and induced the high expression of IL-6, thus activating the expression of p-STAT3, that is, the STAT3 signaling pathway is activated. After the *H. pylori* infection, TMEFF2 induced upregulation of SHP-1 and inhibited STAT3 phosphorylation. After the *H. pylori* infection, FGFR4 was highly expressed, which upregulated SRC and activated STAT signaling pathway. After the *H. pylori* infection, HF-1 interacts with phosphorylated STAT-3, resulting in the suspension of HSP70 expression. After the *H. pylori* infection, VCAM1 can be upregulated by activating the JAK/STAT1 signaling pathway, and then interact with integrin αvβ1/5 to promote tumor invasion. After the activation of STAT signaling pathway, IRF9 can interact with homologous dimers formed by STAT1 and STAT2 on functional IFN stimulatory regulatory elements (ISRE) in the nucleus to regulate the transcription and expression of ISGs.

High FGFR4 expression and STAT3 activation levels can result from the *H. pylori* infection. SRC serves as a bridge between STAT3 and FGFR4, indicating that STAT3 is involved in the stimulation of FGFR4 signaling and demonstrating a positive feedback loop between STAT3 and FGFR4 (Zhang et al., 2022b). Heat shock factor 1 (HSF-1) and phosphorylated STAT-3 interact at the protein level after the *H. pylori* infection of GC cells to produce transcriptionally inactivated HSF-1/STATs complex, which inhibits HSP70 expression, loses its cytoprotective function, and becomes less vulnerable to apoptosis induction (Pierzchalski et al., 2006). A significant stromal component of many types of malignancies are cancer-associated fibroblasts (CAFs) (Su et al., 2018), which express smooth muscle actin (α-SMA), fibroblast activating protein, and fibroblast specific protein 1 (FSP-1) (Kalluri, 2016). By triggering the JAK/STAT1 signaling system, a gastric the *H. pylori* infection can increase the expression of vascular adhesion molecule 1 (VCAM1) in fibroblasts. By connecting with integrin αVβ1/5, VCAM1 can facilitate GC cells’ infiltration into tumors (Shen et al., 2020) (Figure 3).

## *Helicobacter pylori* infection induces host cell apoptosis

An active physiological process of cell death is known as apoptosis. By releasing virulence factors, the *H. pylori* infection can activate and regulate the associated proteins, leading to the death of gastric epithelial cells (Steller, 1995; Ashktorab et al., 2008). Caspases are the aspartate cysteine protease family’s proteasomes, and their activation is typically required for apoptosis to occur (Cohen, 1997), caspases -3, -6, -8, -9, and -10, for example, like caspase-3 or caspase-6, caspase-8 is a promoter protease (Sun et al., 2006). The Bcl-2 family is also a sizable collection of proteins linked to apoptosis. In response to varied stimuli, the balance of pro- and anti-apoptotic molecules determines whether the cells survive or perish. Among them, Bcl-2, Bax, and Bcl-XL control the essential proteins of cell death, mostly through influencing mitochondrial function and encouraging the release of cytochrome C (Guo et al., 2019; Li et al., 2020; Wu et al., 2020; Yi et al., 2020; Liu et al., 2021). *H. pylori* can regulate the downstream apoptotic factor caspase and the expression of related proteins of apoptosis-related genes p53, Bax, and Fas pathways by activating mitochondria to release cytochrome, triggering the expression of various cell substrates and leading to cell apoptosis by interacting with death receptors in the serous membrane.

Reactive oxygen species (ROS) can cause Ape-1, a multifunctional protein that regulates apoptosis, to be produced (Ramana et al., 1998). In the exogenous pathway leading to the gastric epithelial cells’ programmed death after the *H. pylori* infection, APE-1 acetylation is a critical component. Acetylation mutants overexpressing APE-1 prevented caspase-9 activation after the *H. pylori* infection, resulting in decreased expression. The ability of the caspase-8-mediated apoptotic pathway was diminished concurrently (Chattopadhyay et al., 2010). The src-and C-Abl (non-receptor tyrosine kinases)-mediated phosphorylation of CagA is necessary for an *H. pylori*-mediated cell infection (Poppe et al., 2007; Mueller et al., 2012), patients with *H. pylori*-associated gastritis had significantly higher levels of C-Abl in the gastric epithelium and gland. PKC has the ability to directly phosphorylate pAblT735 in gastric epithelial cells. The 14-3-3 protein binds to C-Abl, pushing it to localize in the cytoplasm and inhibiting lowering the production of caspase-8 and caspase-9, blocking the intrinsic apoptotic pathway, and the caspase promoter (Raina et al., 2005; Maiani et al., 2011; Posselt et al., 2019). Patients who have been diagnosed with *H. pylori* have higher levels of IL-18 and IFN-γ, IFN-γ promotes the cellular synthesis of IL-18, by boosting the production of caspase-3, the intracellular cysteine protease ICE (caspase-1) contributes to the processing of IL-18’s active form and to the induction of apoptosis in gastric epithelial cells (Shimada et al., 2008; Koch and Muller, 2015). TRAF1, a member of the TRAF family, interacts with various tumor necrosis factor receptors (TNFR) directly or indirectly and inhibits apoptosis in cells by triggering inflammatory pathways. TRAF1 upregulation can be brought on in gastric epithelial cells by *H. pylori*. The virulence factor CagA can prevent the lysis of TRAF1 and the activation of caspase-8 to TRAF1 following the *H. pylori* infection. Consequently, *H. pylori* can persist in the *H. pylori* can therefore persist in the stomach mucosa for a long time without leading to apoptosis (Wan et al., 2016). By inhibiting FLIP, promoting DISC (death signal inducing complex) assembly by FLIP, activating caspase-8, transmitting the apoptotic signal to the mitochondria, and inducing the release of cytochrome C from the mitochondria into the cytoplasm, *H. pylori* induced TRAIL (tumor necrosis factor-associated apoptosis-inducing ligand) apoptosis signal. Apoptosis resistance can break down when the mitochondrial downstream caspase cascade caspase-9 is triggered (Lin et al., 2014).

After the *H. pylori* infection, corticosteroids, which activate the actin-related protein complex ARP2/3, can help the acid activate the apoptotic function in gastric epithelial cells (VacA), which in turn causes the pro-apoptotic protein Bax to be induced and the anti-apoptotic protein Bcl-2 to be inhibited. This finally causes target cells to undergo apoptosis (Chang et al., 2016). Heat shock proteins (HSPs) serve as molecular chaperones that help damaged proteins refold and fold freshly generated cellular proteins. The GC epithelial cells of *H. pylori* are directly affected by the production of CagA and vacA, increasing their fragility and suppressing the expression of HSP70. On the other hand, the balance between Bax and Bcl-2 expression was altered by the downregulation of HSP70 and the absence of its protective effect on the cell defense system.

Additionally, at the same time, Bcl-2, an anti-apoptotic protein, showed decreased expression (Pierzchalski et al., 2006; Targosz et al., 2012). Through pathways that depend on VDAC and the Bcl-2 family and cytochrome C, the *H. pylori* virulence factor VacA induces caspase-3, causing the activation of caspase-3 and the execution of apoptosis (Lan et al., 2010).

## *Helicobacter pylori* infection induces DNA methylation in host cells

The most extensively researched epigenetic alteration, DNA methylation, has two primary modification mechanisms. The first step is to modify the cytosine residue of the CpG dinucleotide by adding a methyl group to its fifth carbon (Feinberg and Tycko, 2004). The second is the gc-rich region of the genome’s CpG dinucleotide cluster CpG island (CGI), where abnormal methylation results in transcriptional silence and alters the expression of downstream genes (Qu et al., 2013; Usui et al., 2021). Two DNA methylation transferases (DNMTs) catalyze cytosine methylation (Jones and Baylin, 2002), including DNMT3 family methylases, which participate in *de novo* methylation, and DNMT1, which maintains methylation by methylating newly synthesized DNA strands (Costello and Plass, 2001; Hashimoto et al., 2010). One of the primary causes of carcinogenesis is the methylation-induced silence of tumor suppressor genes (Vogelstein et al., 2013).

Connexins Cx32 and Cx43 connect the space between gastric epithelial cells, and the *H. pylori* infection may result in high levels of methylation of their promoters, which lowers their expression. This inhibits the intercellular communication (GJIC) function of the gastric space junction, which causes GC. The expression of Cx32 and Cx43 decreased during the *H. pylori* infection’s chronic atrophic gastritis stage (Wang Y et al., 2014). In GC, FOXD3 is a tumor suppressor that has been epigenetically silenced. Once a FOXD3 promoter methylation is induced at the transcription start point following the *H. pylori* infection. The tumor suppressor role of FOXD3 in GC was later identified, and its direct transcriptional targets CYFIP2 and RARB may act as a conduit for this role. In human GC, FOXD3 prevents the tumor cascade from being downregulated (Cheng et al., 2013). Mucous metaplasia, or TFF2, primarily arises from the stomach’s bottom. The peptide for muscle crack is known as express solution IM (Soutto et al., 2015). The main mechanism of the *H. pylori* infection in chronic TFF2 promoter methylation-induced GC cells directly, primarily in the transcription start site of overlapping CpG dinucleotide, is infected cells after startup TFF2 methylation. Consequently, TFF2 expression declines over time (Peterson et al., 2010). A key regulator of the production of autophagosomes is the microtubule-associated protein 1 light chain 3 (MAP1LC3/LC3) (Ravikumar et al., 2010). MAP1LC3Av1 methylation silencing, which is mostly controlled by DNA methylation in its promoter region, can result from a long-term infection of gastric epithelial cells with *H. pylori*. This can result in an autophagy pathway of cell carcinogenesis. These findings imply that preventing GC brought on by *H. pylori*-associated epigenetic autophagy damage can be accomplished by utilizing demethylating drugs (Muhammad et al., 2017).

## Discussion

The modulation of host proteins in gastric epithelial cells infected with *H. pylori* has been the subject of several investigations. The primary processes of signaling pathways are reviewed in this article, as well as how signaling pathways control the expression of host proteins in GC. For instance, secreted virulence factors regulate the expression of downstream target proteins, activate NF-κB, ERK/MAPK, JAK/STAT, and other signaling pathways or cytokine receptors, enhance or inhibit the inflammatory response after infection, and promote the proliferation and metastasis of GC. Following the *H. pylori* infection, the expression of associated proteins and inflammatory factors is either up- or downregulated, activating the intracellular apoptosis program, starting or stopping the expression of apoptotic proteins, and controlling the associated GC process. Additionally, the *H. pylori* infection can result in DNA methylation, which silences the associated tumor suppressor genes and promotes the growth and spread of cancer.

Despite a thorough examination of the literature, the relevant signaling pathways—such as the PI3K-AKT pathway, Wnt/β-Catenin signaling network, and TGF-β signaling pathway—presented are not all-inclusive. It is vital to pay attention to the linked host proteins because they influence the onset and prognosis of gastric illnesses brought on by the *H. pylori* infections.

## References

[B1] Aaronson D. S. Horvath C. M. (2002). A road map for those who don't know JAK-STAT. Science 296 (5573), 1653–1655. 10.1126/science.1071545 12040185

[B2] Aihara M. Tsuchimoto D. Takizawa H. Azuma A. Wakebe H. Ohmoto Y. (1997). Mechanisms involved in Helicobacter pylori-induced interleukin-8 production by a gastric cancer cell line, MKN45. Infect. Immun. 65 (8), 3218–3224. 10.1128/IAI.65.8.3218-3224.1997 9234778 PMC175455

[B3] Ashktorab H. Dashwood R. H. Dashwood M. M. Zaidi S. I. Hewitt S. M. Green W. R. (2008). *H. pylori*-induced apoptosis in human gastric cancer cells mediated via the release of apoptosis-inducing factor from mitochondria. Helicobacter 13 (6), 506–517. 10.1111/j.1523-5378.2008.00646.x 19166416 PMC7322629

[B4] Backert S. Tegtmeyer N. Fischer W. (2015). Composition, structure and function of the *Helicobacter pylori* cag pathogenicity island encoded type IV secretion system. Future Microbiol. 10 (6), 955–965. 10.2217/fmb.15.32 26059619 PMC4493163

[B5] Bae S. Lim J. W. Kim H. (2021). β-Carotene inhibits expression of matrix metalloproteinase-10 and invasion in *Helicobacter pylori*-infected gastric epithelial cells. Molecules 26 (6), 1567. 10.3390/molecules26061567 33809289 PMC8002206

[B6] Bebb J. R. Letley D. P. Thomas R. J. Aviles F. Collins H. M. Watson S. A. (2003). *Helicobacter pylori* upregulates matrilysin (MMP-7) in epithelial cells *in vivo* and *in vitro* in a Cag dependent manner. Gut 52 (10), 1408–1413. 10.1136/gut.52.10.1408 12970131 PMC1773843

[B7] Boengler K. Hilfiker-Kleiner D. Drexler H. Heusch G. Schulz R. (2008). The myocardial JAK/STAT pathway: From protection to failure. Pharmacol. Ther. 120 (2), 172–185. 10.1016/j.pharmthera.2008.08.002 18786563

[B8] Brandt S. Kwok T. Hartig R. König W. Backert S. (2005). NF-kappaB activation and potentiation of proinflammatory responses by the *Helicobacter pylori* CagA protein. Proc. Natl. Acad. Sci. U. S. A. 102 (26), 9300–9305. 10.1073/pnas.0409873102 15972330 PMC1166591

[B9] Cai B. Cai J. P. Luo Y. L. Chen C. Zhang S. (2015). The specific roles of JAK/STAT signaling pathway in sepsis. Inflammation 38 (4), 1599–1608. 10.1007/s10753-015-0135-z 25676437

[B10] Chang H. Chen D. Ni B. Zuo Q. Wang C. Han R. (2016). Cortactin mediates apoptosis of gastric epithelial cells induced by VacA protein of *Helicobacter pylori* . Dig. Dis. Sci. 61 (1), 80–90. 10.1007/s10620-015-3836-0 26289258

[B11] Chattopadhyay R. Bhattacharyya A. Crowe S. E. (2010). Dual regulation by apurinic/apyrimidinic endonuclease-1 inhibits gastric epithelial cell apoptosis during *Helicobacter pylori* infection. Cancer Res. 70 (7), 2799–2808. 10.1158/0008-5472.CAN-09-4136 20332233 PMC2848894

[B12] Chen G. Y. Shu Y. C. Chuang D. Y. Wang Y. C. (2016). Inflammatory and apoptotic regulatory activity of Tanshinone IIA in Helicobacter pylori-infected cells. Am. J. Chin. Med. 44 (6), 1187–1206. 10.1142/S0192415X1650066X 27627918

[B13] Chen G. Tang N. Wang C. Xiao L. Yu M. Zhao L. (2017). TNF-alpha-inducing protein of *Helicobacter pylori* induces epithelial-mesenchymal transition (EMT) in gastric cancer cells through activation of IL-6/STAT3 signaling pathway. Biochem. Biophys. Res. Commun. 484 (2), 311–317. 10.1016/j.bbrc.2017.01.110 28130110

[B14] Chen Y. Sheppard D. Dong X. Hu X. Chen M. Chen R. (2020). *H. pylori* infection confers resistance to apoptosis via Brd4-dependent BIRC3 eRNA synthesis. Cell. Death Dis. 11 (8), 667. 10.1038/s41419-020-02894-z 32820150 PMC7441315

[B15] Cheng A. S. Li M. S. Kang W. Cheng V. Y. Chou J. L. Lau S. S. (2013). *Helicobacter pylori* causes epigenetic dysregulation of FOXD3 to promote gastric carcinogenesis. Gastroenterology 144 (1), 122–133. 10.1053/j.gastro.2012.10.002 23058321

[B16] Cohen G. M. (1997). Caspases: The executioners of apoptosis. Biochem. J. 326, 1–16. 10.1042/bj3260001 9337844 PMC1218630

[B17] Costa V. L. Henrique R. Danielsen S. A. Duarte-Pereira S. Eknaes M. Skotheim R. I. (2010). Three epigenetic biomarkers, GDF15, TMEFF2, and VIM, accurately predict bladder cancer from DNA-based analyses of urine samples. Clin. Cancer Res. 16 (23), 5842–5851. 10.1158/1078-0432.CCR-10-1312 20975101

[B18] Costa A. M. Ferreira R. M. Pinto-Ribeiro I. Sougleri I. S. Oliveira M. J. Carreto L. (2016). *Helicobacter pylori* activates matrix metalloproteinase 10 in gastric epithelial cells via EGFR and ERK-mediated pathways. J. Infect. Dis. 213 (11), 1767–1776. 10.1093/infdis/jiw031 26802142

[B19] Costello J. F. Plass C. (2001). Methylation matters. J. Med. Genet. 38 (5), 285–303. 10.1136/jmg.38.5.285 11333864 PMC1734882

[B20] Cover T. L. Blanke S. R. (2005). *Helicobacter pylori* VacA, a paradigm for toxin multifunctionality. Nat. Rev. Microbiol. 3 (4), 320–332. 10.1038/nrmicro1095 15759043

[B21] Crowe S. E. (2019). *Helicobacter pylori* infection. N. Engl. J. Med. 380 (12), 1158–1165. 10.1056/NEJMcp1710945 30893536

[B22] Dickson J. H. Grabowska A. El-Zaatari M. Atherton J. Watson S. A. (2006). *Helicobacter pylori* can induce heparin-binding epidermal growth factor expression via gastrin and its receptor. Cancer Res. 66 (15), 7524–7531. 10.1158/0008-5472.CAN-05-3246 16885350

[B23] Ding S. Z. Smith M. F. Jr. Goldberg J. B. (2008). *Helicobacter pylori* and mitogen-activated protein kinases regulate the cell cycle, proliferation and apoptosis in gastric epithelial cells. J. Gastroenterol. Hepatol. 23, e67–e78. 10.1111/j.1440-1746.2007.04912.x 18702686

[B24] Feinberg A. P. Tycko B. (2004). The history of cancer epigenetics. Nat. Rev. Cancer 4 (2), 143–153. 10.1038/nrc1279 14732866

[B25] Fischer W. Püls J. Buhrdorf R. Gebert B. Odenbreit S. Haas R. (2001). Systematic mutagenesis of the *Helicobacter pylori* cag pathogenicity island: Essential genes for CagA translocation in host cells and induction of interleukin-8. Mol. Microbiol. 42 (5), 1337–1348. 10.1046/j.1365-2958.2001.02714.x 11886563

[B26] Gao J. P. Xu W. Liu W. T. Yan M. Zhu Z. G. (2018). Tumor heterogeneity of gastric cancer: From the perspective of tumor-initiating cell. World J. Gastroenterol. 24 (24), 2567–2581. 10.3748/wjg.v24.i24.2567 29962814 PMC6021770

[B27] Gemel J. Simon A. R. Patel D. Xu Q. Matiukas A. Veenstra R. D. (2014). Degradation of a connexin40 mutant linked to atrial fibrillation is accelerated. J. Mol. Cell. Cardiol. 74, 330–339. 10.1016/j.yjmcc.2014.06.010 24973497 PMC4135452

[B28] Göõz M. Göõz P. Smolka A. J. (2001). Epithelial and bacterial metalloproteinases and their inhibitors in *H. pylori* infection of human gastric cells. Am. J. Physiol. Gastrointest. Liver Physiol. 281 (3), G823–G832. 10.1152/ajpgi.2001.281.3.G823 11518695

[B29] Guo Q. Jing F. J. Qu H. J. Xu W. Han B. Xing X. M. (2019). Ubenimex reverses MDR in gastric cancer cells by activating caspase-3-mediated apoptosis and suppressing the expression of membrane transport proteins. Biomed. Res. Int. 2019, 4390839. 10.1155/2019/4390839 30915355 PMC6402206

[B30] Guo Y. Zhang T. Shi Y. Zhang J. Li M. Lu F. (2020a). *Helicobacter pylori* inhibits GKN1 expression via the CagA/p-ERK/AUF1 pathway. Helicobacter 25 (1), e12665. 10.1111/hel.12665 31657090

[B31] Guo Y. Pan W. W. Liu S. B. Shen Z. F. Xu Y. Hu L. L. (2020b). ERK/MAPK signalling pathway and tumorigenesis. Exp. Ther. Med. 19 (3), 1997–2007. 10.3892/etm.2020.8454 32104259 PMC7027163

[B32] Hashimoto H. Vertino P. M. Cheng X. (2010). Molecular coupling of DNA methylation and histone methylation. Epigenomics 2 (5), 657–669. 10.2217/epi.10.44 21339843 PMC3039846

[B33] Hayden M. S. Ghosh S. (2011). NF-κB in immunobiology. Cell. Res. 21 (2), 223–244. 10.1038/cr.2011.13 21243012 PMC3193440

[B34] Hojo M. Miwa H. Kikuchi S. Sato N. (2000). Do mucosal defensive agents improve the cure rate when used with dual or triple therapy regimens for eradicating *Helicobacter pylori* infection? Aliment. Pharmacol. Ther. 14 (2), 193–201. 10.1046/j.1365-2036.2000.00692.x 10651660

[B35] Jiang H. Zhou Y. Liao Q. Ouyang H. (2014). *Helicobacter pylori* infection promotes the invasion and metastasis of gastric cancer through increasing the expression of matrix metalloproteinase-1 and matrix metalloproteinase-10. Exp. Ther. Med. 8 (3), 769–774. 10.3892/etm.2014.1822 25120597 PMC4113550

[B36] Jones P. A. Baylin S. B. (2002). The fundamental role of epigenetic events in cancer. Nat. Rev. Genet. 3 (6), 415–428. 10.1038/nrg816 12042769

[B37] Jove R. (2000). Preface: STAT signaling. Oncogene 19 (21), 2466–2467. 10.1038/sj.onc.1203549 10851044

[B38] Kalluri R. (2016). The biology and function of fibroblasts in cancer. Nat. Rev. Cancer 16 (9), 582–598. 10.1038/nrc.2016.73 27550820

[B39] Katz M. Amit I. Yarden Y. (2007). Regulation of MAPKs by growth factors and receptor tyrosine kinases. Biochim. Biophys. Acta 1773 (8), 1161–1176. 10.1016/j.bbamcr.2007.01.002 17306385 PMC2758354

[B40] Khanna P. Chua P. J. Bay B. H. Baeg G. H. (2015). The JAK/STAT signaling cascade in gastric carcinoma (Review). Int. J. Oncol. 47 (5), 1617–1626. 10.3892/ijo.2015.3160 26398764

[B41] Kimura M. Goto S. Ihara Y. Wada A. Yahiro K. Niidome T. (2001). Impairment of glutathione metabolism in human gastric epithelial cells treated with vacuolating cytotoxin from *Helicobacter pylori* . Microb. Pathog. 31 (1), 29–36. 10.1006/mpat.2001.0446 11427034

[B42] Koch K. N. Muller A. (2015). *Helicobacter pylori* activates the TLR2/NLRP3/caspase-1/IL-18 axis to induce regulatory T-cells, establish persistent infection and promote tolerance to allergens. Gut Microbes 6 (6), 382–387. 10.1080/19490976.2015.1105427 26727421 PMC4826104

[B43] Krueger S. Hundertmark T. Kalinski T. Peitz U. Wex T. Malfertheiner P. (2006). *Helicobacter pylori* encoding the pathogenicity island activates matrix metalloproteinase 1 in gastric epithelial cells via JNK and ERK. J. Biol. Chem. 281 (5), 2868–2875. 10.1074/jbc.M511053200 16321971

[B44] Lan C. H. Sheng J. Q. Fang D. C. Meng Q. Z. Fan L. L. Huang Z. R. (2010). Involvement of VDAC1 and Bcl-2 family of proteins in VacA-induced cytochrome c release and apoptosis of gastric epithelial carcinoma cells. J. Dig. Dis. 11 (1), 43–49. 10.1111/j.1751-2980.2009.00412.x 20132430

[B45] Li R. Zou X. Zhu T. Xu H. Li X. Zhu L. (2020). Destruction of neutrophil extracellular traps promotes the apoptosis and inhibits the invasion of gastric cancer cells by regulating the expression of bcl-2, Bax and NF-κB. OncoTargets Ther. Vol. 13, 5271–5281. 10.2147/OTT.S227331< PMC7293391 32606746

[B46] Lin K. Taylor J. R. Jr. Wu T. D. Gutierrez J. Elliott J. M. Vernes J. M. (2011). TMEFF2 is a PDGF-AA binding protein with methylation-associated gene silencing in multiple cancer types including glioma. PLoS One 6 (4), e18608. 10.1371/journal.pone.0018608 21559523 PMC3084709

[B47] Lin W. C. Tsai H. F. Liao H. J. Tang C. H. Wu Y. Y. Hsu P. I. (2014). *Helicobacter pylori* sensitizes TNF-related apoptosis-inducing ligand (TRAIL)-mediated apoptosis in human gastric epithelial cells through regulation of FLIP. Cell. Death Dis. 5, e1109. 10.1038/cddis.2014.81 24603337 PMC3973194

[B48] Liu F. Zhang W. Yang F. Feng T. Zhou M. Yu Y. (2016). Interleukin-6-stimulated progranulin expression contributes to the malignancy of hepatocellular carcinoma cells by activating mTOR signaling. Sci. Rep. 6, 21260. 10.1038/srep21260 26879559 PMC4754634

[B49] Liu T. Zhang L. Joo D. Sun S. C. (2017). NF-kappaB signaling in inflammation. Signal Transduct. Target Ther. 2, 17023. 10.1038/sigtrans.2017.23 29158945 PMC5661633

[B50] Liu J. F. Guo D. Kang E. M. Wang Y. S. Gao X. Z. Cong H. Y. (2021). Acute and chronic infection of *H. pylori* caused the difference in apoptosis of gastric epithelial cells. Microb. Pathog. 150, 104717. 10.1016/j.micpath.2020.104717 33421608

[B51] Machlowska J. Baj J. Sitarz M. Maciejewski R. Sitarz R. (2020). Gastric cancer: Epidemiology, risk factors, classification, genomic characteristics and treatment strategies. Int. J. Mol. Sci. 21 (11), 4012. 10.3390/ijms21114012 32512697 PMC7312039

[B52] Maeda S. Yoshida H. Ogura K. Mitsuno Y. Hirata Y. Yamaji Y. (2000). *H. pylori* activates NF-kappaB through a signaling pathway involving IkappaB kinases, NF-kappaB-inducing kinase, TRAF2, and TRAF6 in gastric cancer cells. Gastroenterology 119 (1), 97–108. 10.1053/gast.2000.8540 10889159

[B53] Maiani E. Diederich M. Gonfloni S. (2011). DNA damage response: The emerging role of c-abl as a regulatory switch? Biochem. Pharmacol. 82 (10), 1269–1276. 10.1016/j.bcp.2011.07.001 21763684

[B54] Maubach G. Sokolova O. Wolfien M. Rothkotter H. J. Naumann M. (2013). Ca2+/calmodulin-dependent kinase II contributes to inhibitor of nuclear factor-kappa B kinase complex activation in *Helicobacter pylori* infection. Int. J. Cancer 133 (6), 1507–1512. 10.1002/ijc.28148 23463379

[B55] Maubach G. Lim M. C. C. Sokolova O. Backert S. Meyer T. F. Naumann M. (2021). TIFA has dual functions in Helicobacter pylori-induced classical and alternative NF-κB pathways. EMBO Rep. 22 (9), e52878. 10.15252/embr.202152878 34328245 PMC8419686

[B56] Maubach G. Vieth M. Boccellato F. Naumann M. (2022). Helicobacter pylori-induced NF-κB: Trailblazer for gastric pathophysiology. Trends Mol. Med. 28 (3), 210–222. 10.1016/j.molmed.2021.12.005 35012886

[B57] Mejias-Luque R. Zoller J. Anderl F. Loew-Gil E. Vieth M. Adler T. (2017). Lymphotoxin beta receptor signalling executes Helicobacter pylori-driven gastric inflammation in a T4SS-dependent manner. Gut 66 (8), 1369–1381. 10.1136/gutjnl-2015-310783 27196595

[B58] Mori N. Sato H. Hayashibara T. Senba M. Geleziunas R. Wada A. (2003). *Helicobacter pylori* induces matrix metalloproteinase-9 through activation of nuclear factor kappaB. Gastroenterology 124 (4), 983–992. 10.1053/gast.2003.50152 12671895

[B59] Mueller D. Tegtmeyer N. Brandt S. Yamaoka Y. De Poire E. Sgouras D. (2012). c-Src and c-Abl kinases control hierarchic phosphorylation and function of the CagA effector protein in Western and East Asian *Helicobacter pylori* strains. J. Clin. Invest. 122 (4), 1553–1566. 10.1172/JCI61143 22378042 PMC3314471

[B60] Muhammad J. S. Nanjo S. Ando T. Yamashita S. Maekita T. Ushijima T. (2017). Autophagy impairment by Helicobacter pylori-induced methylation silencing of MAP1LC3Av1 promotes gastric carcinogenesis. Int. J. Cancer 140 (10), 2272–2283. 10.1002/ijc.30657 28214334

[B61] Muller A. (2012). Multistep activation of the *Helicobacter pylori* effector CagA. J. Clin. Invest. 122 (4), 1192–1195. 10.1172/JCI61578 22378039 PMC3314475

[B62] Neumann M. Naumann M. (2007). Beyond IkappaBs: Alternative regulation of NF-kappaB activity. FASEB J. 21 (11), 2642–2654. 10.1096/fj.06-7615rev 17431096

[B63] Ng G. Z. Menheniott T. R. Every A. L. Stent A. Judd L. M. Chionh Y. T. (2016). The MUC1 mucin protects against *Helicobacter pylori* pathogenesis in mice by regulation of the NLRP3 inflammasome. Gut 65 (7), 1087–1099. 10.1136/gutjnl-2014-307175 26079943

[B64] Noto J. M. Peek R. M. Jr (2012). *Helicobacter pylori*: An overview. Methods Mol. Biol. 921, 7–10. 10.1007/978-1-62703-005-2_2 23015485

[B65] Odenbreit S. Puls J. Sedlmaier B. Gerland E. Fischer W. Haas R. (2000). Translocation of *Helicobacter pylori* CagA into gastric epithelial cells by type IV secretion. Science 287 (5457), 1497–1500. 10.1126/science.287.5457.1497 10688800

[B66] Ogasa M. Miyazaki Y. Hiraoka S. Kitamura S. Nagasawa Y. Kishida O. (2003). Gastrin activates nuclear factor kappaB (NFkappaB) through a protein kinase C dependent pathway involving NFkappaB inducing kinase, inhibitor kappaB (IkappaB) kinase, and tumour necrosis factor receptor associated factor 6 (TRAF6) in MKN-28 cells transfected with gastrin receptor. Gut 52 (6), 813–819. 10.1136/gut.52.6.813 12740336 PMC1773663

[B67] Palframan S. L. Kwok T. Gabriel K. (2012). Vacuolating cytotoxin A (VacA), a key toxin for *Helicobacter pylori* pathogenesis. Front. Cell. Infect. Microbiol. 2, 92. 10.3389/fcimb.2012.00092 22919683 PMC3417644

[B68] Pathak S. K. Tavares R. de Klerk N. Spetz A. L. Jonsson A. B. (2013). *Helicobacter pylori* protein JHP0290 binds to multiple cell types and induces macrophage apoptosis via tumor necrosis factor (TNF)-dependent and independent pathways. PLoS One 8 (11), e77872. 10.1371/journal.pone.0077872 24223737 PMC3815203

[B69] Peng C. Ouyang Y. Lu N. Li N. (2020). The NF-κB signaling pathway, the microbiota, and gastrointestinal tumorigenesis: Recent advances. Front. Immunol. 11, 1387. 10.3389/fimmu.2020.01387 32695120 PMC7338561

[B70] Perrais M. Rousseaux C. Ducourouble M. P. Courcol R. Vincent P. Jonckheere N. (2014). *Helicobacter pylori* urease and flagellin alter mucin gene expression in human gastric cancer cells. Gastric Cancer 17 (2), 235–246. 10.1007/s10120-013-0267-5 23703470

[B71] Peterson A. J. Menheniott T. R. O'Connor L. Walduck A. K. Fox J. G. Kawakami K. (2010). *Helicobacter pylori* infection promotes methylation and silencing of trefoil factor 2, leading to gastric tumor development in mice and humans. Gastroenterology 139 (6), 2005–2017. 10.1053/j.gastro.2010.08.043 20801119 PMC3970568

[B72] Petryszyn P. Chapelle N. Matysiak-Budnik T. (2020). Gastric cancer: Where are we heading? Dig. Dis. 38 (4), 280–285. 10.1159/000506509 32062657

[B73] Pierzchalski P. Krawiec A. Ptak-Belowska A. Barańska A. Konturek S. J. Pawlik W. W. (2006). The mechanism of heat-shock protein 70 gene expression abolition in gastric epithelium caused by *Helicobacter pylori* infection. Helicobacter 11 (2), 96–104. 10.1111/j.1523-5378.2006.00383.x 16579839

[B74] Piñeiro M. Alava M. A. González-Ramón N. Osada J. Lasierra P. Larrad L. (1999). ITIH4 serum concentration increases during acute-phase processes in human patients and is up-regulated by interleukin-6 in hepatocarcinoma HepG2 cells. Biochem. Biophys. Res. Commun. 263 (1), 224–229. 10.1006/bbrc.1999.1349 10486281

[B75] Plotnikov A. Zehorai E. Procaccia S. Seger R. (2011). The MAPK cascades: Signaling components, nuclear roles and mechanisms of nuclear translocation. Biochim. Biophys. Acta 1813 (9), 1619–1633. 10.1016/j.bbamcr.2010.12.012 21167873

[B76] Poppe M. Feller S. M. Romer G. Wessler S. (2007). Phosphorylation of *Helicobacter pylori* CagA by c-Abl leads to cell motility. Oncogene 26 (24), 3462–3472. 10.1038/sj.onc.1210139 17160020

[B77] Posselt G. Wiesauer M. Chichirau B. E. Engler D. Krisch L. M. Gadermaier G. (2019). Helicobacter pylori-controlled c-Abl localization promotes cell migration and limits apoptosis. Cell. Commun. Signal 17 (1), 10. 10.1186/s12964-019-0323-9 30704478 PMC6357398

[B78] Qu Y. Dang S. Hou P. (2013). Gene methylation in gastric cancer. Clin. Chim. Acta 424, 53–65. 10.1016/j.cca.2013.05.002 23669186

[B79] Raina D. Pandey P. Ahmad R. Bharti A. Ren J. Kharbanda S. (2005). c-Abl tyrosine kinase regulates caspase-9 autocleavage in the apoptotic response to DNA damage. J. Biol. Chem. 280 (12), 11147–11151. 10.1074/jbc.M413787200 15657060

[B80] Raju D. Hussey S. Ang M. Terebiznik M. R. Sibony M. Galindo-Mata E. (2012). Vacuolating cytotoxin and variants in Atg16L1 that disrupt autophagy promote *Helicobacter pylori* infection in humans. Gastroenterology 142 (5), 1160–1171. 10.1053/j.gastro.2012.01.043 22333951 PMC3336037

[B81] Ramana C. V. Boldogh I. Izumi T. Mitra S. (1998). Activation of apurinic/apyrimidinic endonuclease in human cells by reactive oxygen species and its correlation with their adaptive response to genotoxicity of free radicals. Proc. Natl. Acad. Sci. U. S. A. 95 (9), 5061–5066. 10.1073/pnas.95.9.5061 9560228 PMC20213

[B82] Ravikumar B. Sarkar S. Davies J. E. Futter M. Garcia-Arencibia M. Green-Thompson Z. W. (2010). Regulation of mammalian autophagy in physiology and pathophysiology. Physiol. Rev. 90 (4), 1383–1435. 10.1152/physrev.00030.2009 20959619

[B83] Rawlings J. S. Rosler K. M. Harrison D. A. (2004). The JAK/STAT signaling pathway. J. Cell. Sci. 117 (8), 1281–1283. 10.1242/jcs.00963 15020666

[B84] Sabio G. Davis R. J. (2014). TNF and MAP kinase signalling pathways. Seminars Immunol. 26 (3), 237–245. 10.1016/j.smim.2014.02.009 PMC4099309 24647229

[B85] Sakitani K. Hirata Y. Hayakawa Y. Serizawa T. Nakata W. Takahashi R. (2012). Role of interleukin-32 in Helicobacter pylori-induced gastric inflammation. Infect. Immun. 80 (11), 3795–3803. 10.1128/IAI.00637-12 22890997 PMC3486038

[B86] Sebkova L. Pellicano A. Monteleone G. Grazioli B. Guarnieri G. Imeneo M. (2004). Extracellular signal-regulated protein kinase mediates interleukin 17 (IL-17)-induced IL-8 secretion in Helicobacter pylori-infected human gastric epithelial cells. Infect. Immun. 72 (9), 5019–5026. 10.1128/IAI.72.9.5019-5026.2004 15321994 PMC517427

[B87] Shaffer C. L. Gaddy J. A. Loh J. T. Johnson E. M. Hill S. Hennig E. E. (2011). *Helicobacter pylori* exploits a unique repertoire of type IV secretion system components for pilus assembly at the bacteria-host cell interface. PLoS Pathog. 7 (9), e1002237. 10.1371/journal.ppat.1002237 21909278 PMC3164655

[B88] Shen J. Zhai J. You Q. Zhang G. He M. Yao X. (2020). Cancer-associated fibroblasts-derived VCAM1 induced by *H. pylori* infection facilitates tumor invasion in gastric cancer. Oncogene 39 (14), 2961–2974. 10.1038/s41388-020-1197-4 32034307

[B89] Shimada M. Ando T. Peek R. M. Watanabe O. Ishiguro K. Maeda O. (2008). *Helicobacter pylori* infection upregulates interleukin-18 production from gastric epithelial cells. Eur. J. Gastroenterol. Hepatol. 20 (12), 1144–1150. 10.1097/MEG.0b013e32830edb15 18946358 PMC3372857

[B90] Soutto M. Romero-Gallo J. Krishna U. Piazuelo M. B. Washington M. K. Belkhiri A. (2015). Loss of TFF1 promotes Helicobacter pylori-induced β-catenin activation and gastric tumorigenesis. Oncotarget 6 (20), 17911–17922. 10.18632/oncotarget.3772 25980439 PMC4627225

[B91] Steller H. (1995). Mechanisms and genes of cellular suicide. Science 267 (5203), 1445–1449. 10.1126/science.7878463 7878463

[B92] Su S. Chen J. Yao H. Liu J. Yu S. Lao L. (2018). CD10(+)GPR77(+) cancer-associated fibroblasts promote cancer formation and chemoresistance by sustaining cancer stemness. Cell. 172 (4), 841–856. 10.1016/j.cell.2018.01.009 29395328

[B93] Suganuma M. Watanabe T. Sueoka E. Lim I. K. Fujiki H. (2021). Role of TNF-α-inducing protein secreted by *Helicobacter pylori* as a tumor promoter in gastric cancer and emerging preventive strategies. Toxins (Basel) 13 (3), 181. 10.3390/toxins13030181 33804551 PMC7999756

[B94] Sun Y. Chen X. Y. Liu J. Cheng X. X. Wang X. W. Kong Q. Y. (2006). Differential caspase-3 expression in noncancerous, premalignant and cancer tissues of stomach and its clinical implication. Cancer Detect Prev. 30 (2), 168–173. 10.1016/j.cdp.2006.02.004 16697119

[B95] Sun T. T. Tang J. Y. Du W. Zhao H. J. Zhao G. Yang S. L. (2015). Bidirectional regulation between TMEFF2 and STAT3 may contribute to Helicobacter pylori-associated gastric carcinogenesis. Int. J. Cancer 136 (5), 1053–1064. 10.1002/ijc.29061 24996057

[B96] Targosz A. Brzozowski T. Pierzchalski P. Szczyrk U. Ptak-Belowska A. Konturek S. J. (2012). *Helicobacter pylori* promotes apoptosis, activates cyclooxygenase (COX)-2 and inhibits heat shock protein HSP70 in gastric cancer epithelial cells. Inflamm. Res. 61 (9), 955–966. 10.1007/s00011-012-0487-x 22610150 PMC3418497

[B97] Tavares R. Pathak S. K. (2015). *Helicobacter pylori* protein JHP0290 exhibits proliferative and anti-apoptotic effects in gastric epithelial cells. PLoS One 10 (4), e0124407. 10.1371/journal.pone.0124407 25879227 PMC4400171

[B98] Usui G. Matsusaka K. Mano Y. Urabe M. Funata S. Fukayama M. (2021). DNA methylation and genetic aberrations in gastric cancer. Digestion 102 (1), 25–32. 10.1159/000511243 33070127

[B99] Vinken M. Vanhaecke T. Papeleu P. Snykers S. Henkens T. Rogiers V. (2006). Connexins and their channels in cell growth and cell death. Cell. Signal 18 (5), 592–600. 10.1016/j.cellsig.2005.08.012 16183253

[B100] Vogelstein B. Papadopoulos N. Velculescu V. E. Zhou S. Diaz L. A. Jr. Kinzler K. W. (2013). Cancer genome landscapes. Science 339 (6127), 1546–1558. 10.1126/science.1235122 23539594 PMC3749880

[B101] Wan X. K. Yuan S. L. Wang Y. C. Tao H. X. Jiang W. Guan Z. Y. (2016). *Helicobacter pylori* inhibits the cleavage of TRAF1 via a CagA-dependent mechanism. World J. Gastroenterol. 22 (48), 10566–10574. 10.3748/wjg.v22.i48.10566 28082808 PMC5192267

[B102] Wang F F. Meng W. Wang B. Qiao L. (2014). Helicobacter pylori-induced gastric inflammation and gastric cancer. Cancer Lett. 345 (2), 196–202. 10.1016/j.canlet.2013.08.016 23981572

[B103] Wang Y Y. Huang L. H. Xu C. X. Xiao J. Zhou L. Cao D. (2014). Connexin 32 and 43 promoter methylation in Helicobacter pylori-associated gastric tumorigenesis. World J. Gastroenterol. 20 (33), 11770–11779. 10.3748/wjg.v20.i33.11770 25206281 PMC4155367

[B104] Wroblewski L. E. Peek R. M. Jr (2016). *Helicobacter pylori*, cancer, and the gastric microbiota. Adv. Exp. Med. Biol. 908, 393–408. 10.1007/978-3-319-41388-4_19 27573782

[B105] Wu X. Zhang Y. Guo J. Yan X. Shen L. Zhou J. (2020). MAC30 knockdown inhibits proliferation and enhance apoptosis of gastric cancer by suppressing wnt/β-cateninsignaling pathway. Gastroenterol. Res. Pract. 2020, 6358685. 10.1155/2020/6358685 32904598 PMC7456481

[B106] Xin P. Xu X. Deng C. Liu S. Wang Y. Zhou X. (2020). The role of JAK/STAT signaling pathway and its inhibitors in diseases. Int. Immunopharmacol. 80, 106210. 10.1016/j.intimp.2020.106210 31972425

[B107] Yahiro K. Akazawa Y. Nakano M. Suzuki H. Hisatune J. Isomoto H. (2015). *Helicobacter pylori* VacA induces apoptosis by accumulation of connexin 43 in autophagic vesicles via a Rac1/ERK-dependent pathway. Cell. Death Discov. 1, 15035. 10.1038/cddiscovery.2015.35 27551466 PMC4979424

[B108] Yasumoto K. Okamoto S. Mukaida N. Murakami S. Mai M. Matsushima K. (1992). Tumor necrosis factor alpha and interferon gamma synergistically induce interleukin 8 production in a human gastric cancer cell line through acting concurrently on AP-1 and NF-kB-like binding sites of the interleukin 8 gene. J. Biol. Chem. 267 (31), 22506–22511. 10.1016/s0021-9258(18)41701-2 1331059

[B109] Yi H. Qiu M. Z. Yuan L. Luo Q. Pan W. Zhou S. (2020). Bcl-2/Bcl-xl inhibitor APG-1252-M1 is a promising therapeutic strategy for gastric carcinoma. Cancer Med. 9 (12), 4197–4206. 10.1002/cam4.3090 32346976 PMC7300393

[B110] Yin Y. Grabowska A. M. Clarke P. A. Whelband E. Robinson K. Argent R. H. (2010). *Helicobacter pylori* potentiates epithelial:mesenchymal transition in gastric cancer: Links to soluble HB-egf, gastrin and matrix metalloproteinase-7. Gut 59 (8), 1037–1045. 10.1136/gut.2009.199794 20584780 PMC2976077

[B111] Yoon H. Kim S. G. Kim B. K. Shin E. Kim N. Lee H. J. (2017). *Helicobacter pylori* eradication downregulates cellular inhibitor of apoptosis protein 2 in gastric carcinogenesis. Gut Liver 11 (1), 79–86. 10.5009/gnl15585 27282269 PMC5221864

[B112] Yu H. Pardoll D. Jove R. (2009). STATs in cancer inflammation and immunity: A leading role for STAT3. Nat. Rev. Cancer 9 (11), 798–809. 10.1038/nrc2734 19851315 PMC4856025

[B113] Zhang Y. Zhou X. Zhang Q. Zhang Y. Wang X. Cheng L. (2019). Involvement of NF-κB signaling pathway in the regulation of PRKAA1-mediated tumorigenesis in gastric cancer. Artif. Cells Nanomed Biotechnol. 47 (1), 3677–3686. 10.1080/21691401.2019.1657876 31841039

[B114] Zhang X. Li C. Chen D. He X. Zhao Y. Bao L. (2022a). *H. pylori* CagA activates the NLRP3 inflammasome to promote gastric cancer cell migration and invasion. Inflamm. Res. 71 (1), 141–155. 10.1007/s00011-021-01522-6 34854954

[B115] Zhang X. Soutto M. Chen Z. Bhat N. Zhu S. Eissmann M. F. (2022b). Induction of fibroblast growth factor receptor 4 by *Helicobacter pylori* via signal transducer and activator of transcription 3 with a feedforward activation loop involving steroid receptor coactivator signaling in gastric cancer. Gastroenterology. 163. 620. 10.1053/j.gastro.2022.05.016 35588797 PMC9629135

[B116] Zhu Y. Zhong X. Zheng S. Du Q. Xu W. (2005). Transformed immortalized gastric epithelial cells by virulence factor CagA of *Helicobacter pylori* through Erk mitogen-activated protein kinase pathway. Oncogene 24 (24), 3886–3895. 10.1038/sj.onc.1208551 15856031

